# Help-Seeking in People with Exceptional Experiences: Results from a General Population Sample

**DOI:** 10.3389/fpubh.2014.00051

**Published:** 2014-05-21

**Authors:** Karin Landolt, Amrei Wittwer, Thomas Wyss, Lui Unterassner, Wolfgang Fach, Peter Krummenacher, Peter Brugger, Helene Haker, Wolfram Kawohl, Pius August Schubiger, Gerd Folkers, Wulf Rössler

**Affiliations:** ^1^Department of Psychiatry, Psychotherapy and Psychosomatics, Zürich University Hospital for Psychiatry, Zurich, Switzerland; ^2^Collegium Helveticum, Zurich, Switzerland; ^3^Institute for Frontier Areas of Psychology and Mental Health, Freiburg, Germany; ^4^Department of Psychology, Clinical Psychology and Psychotherapy, University of Basel, Basel, Switzerland; ^5^Department of Neurology, University Hospital Zurich, Zurich, Switzerland; ^6^Translational Neuromodeling Unit (TNU), Institute for Biomedical Engineering, University of Zurich, Zurich, Switzerland; ^7^Translational Neuromodeling Unit (TNU), Institute for Biomedical Engineering, ETH Zurich, Zurich, Switzerland; ^8^Department of Psychiatry, Psychotherapy and Psychosomatics, Center for Social Psychiatry, Zürich University Hospital for Psychiatry, Zurich, Switzerland; ^9^Leuphana University, Lüneburg, Germany; ^10^Laboratory of Neuroscience (LIM27), Institute of Psychiatry, University of São Paulo, São Paulo, Brazil

**Keywords:** psychiatric disorder, exceptional experiences, help-seeking, public health, epidemiology

## Abstract

**Background:** Exceptional experiences (EE) are experiences that deviate from ordinary experiences, for example precognition, supernatural appearances, or déjà vues. In spite of the high frequency of EE in the general population, little is known about their effect on mental health and about the way people cope with EE. This study aimed to assess the quality and quantity of EE in persons from the Swiss general population, to identify the predictors of their help-seeking, and to determine how many of them approach the mental health system.

**Methods:** An on-line survey was used to evaluate a quota sample of 1580 persons representing the Swiss general population with respect to gender, age, and level of education. Multinomial logistic regression was applied to integrate help-seeking, self-reported mental disorder, and other variables in a statistical model designed to identify predictors of help-seeking in persons with EE.

**Results:** Almost all participants (91%) experienced at least one EE. Generally, help-seeking was more frequent when the EE were of negative valence. Help-seeking because of EE was less frequent in persons without a self-reported mental disorder (8.6%) than in persons with a disorder (35.1%) (OR = 5.7). Even when frequency and attributes of EE were controlled for, people without a disorder sought four times less often help because of EE than expected. Persons with a self-reported diagnosis of mental disorder preferred seeing a mental health professional. Multinomial regression revealed a preference for healers in women with less education, who described themselves as believing and also having had more impressive EE.

**Conclusion:** Persons with EE who do not indicate a mental disorder less often sought help because of EE than persons who indicated a mental disorder. We attribute this imbalance to a high inhibition threshold to seek professional help. Moreover, especially less educated women did not approach the mental health care system as often as other persons with EE, but preferred seeing a healer.

## Introduction

Exceptional or extra-ordinary experiences (EE) are frequent in the population, with the prevalence being estimated at 30%–75% (1, 2). Most people have at least once in their life had EE, like hearing the voices of dead loved ones, precognition, supernatural appearances, or déjà vues. In spite of the high frequency, little is known about their effect on mental health and about the way people cope with EE. Although EE are fascinating, EE with a high negative valence can cause subjective suffering (3), and EE and magical ideation (MI) are conceptually close to psychosis (4, 5). When EE are successfully coped with, they can add to psychological health (3). This indicates that people who are not able to cope with their EE could benefit from easy accessible and professional help. The skills of health professionals and healers in dealing with EE are yet to be evaluated.

Exceptional experiences do not fit yet into the psychopathological classification systems, because the exact interrelation between mental disorders and EE is not known yet. Our definition of EE aims to encompass a wide spectrum of experiences: EE are usually understood to deviate from or being at variance with ordinary experiences, as defined by typical “reality models” (6) that have been adopted by individuals to fit within their own socio-cultural environment (3, 7). In modern societies, such models are largely based on well-founded epistemological concepts (e.g., cause-and-effect relations) and established scientific principles or laws (e.g., gravity). This coarse characterization covers four broad types of phenomena: (1) external phenomenon, such as apparitions, which reflect the environment of an individual, i.e., the “world” model; (2) internal phenomenon, such as invasive thoughts, i.e., the “self” model (6); (3) dissociation phenomenon, which can occur due to deviations in the relations between the “world-model” and “self-model,” such as out-of-body experiences; or (4) coincidence phenomenon, including extrasensory perceptions (7, 8). For individuals undergoing those experiences, the phenomenological typology of EE, which has been strongly inspired by Metzinger’s representational approach within the philosophy of mind (6), is neutral with respect to their consequences. This means that each type of EE can have positive and negative consequences, for example, the quality of life may either decline or improve, individuals may suffer or profit because of them, and those experiences may or may not belong to a category of mental (psychiatric) disorders. This last differentiation is important because symptoms of mental disorders and EE can be quite similar.

Exceptional experiences must be systematically distinguished from the belief in such experiences, e.g., MI (9). Although experiences and beliefs are correlated, individuals with strong beliefs in non-causal influences or unknown physical forces (10) do not necessarily report EE and vice versa. An important distinction between the two is that experiences have more state character while beliefs are predominantly traits. Although there may be trait-like dispositions for EE, they are currently unknown. It is also far from well-established that beliefs show a one-to-one relationship with such dispositions.

Considering EE, the distinction between illness and health seems difficult. It is not yet clear whether EE are part of the subclinical end of the psychosis spectrum (11–13), or whether they belong to another category of phenomena. How EE can be separated from or are interrelated with schizotypal signs as described by Johns and van Os (11) remains unclear yet. Both EE and magical beliefs have found to be associated with phases of mental disorder (9, 14, 15). However, neither do all persons who report EE also suffer from mental disorders, nor are all experiences unpleasant. A recent continuum hypothesis (11–13) has postulated a gradual change between mental health and disorder, which has been argued to be more plausible than a discrete, absolute assignment to just one of those two categories. According to this hypothesis, it is self-evident that many EE are not indicators of mental disorders, at least when they are few. Results from neurophysiological evaluations have supported this continuum hypothesis. For example, dopaminergic hyperactivity and increased dopamine availability seem to be involved along the entire schizophrenia spectrum (16–20), and both also coincide with high scores in MI (21). Furthermore, MI might have some health-promoting aspects, helping a person cope with life events (3) or high dopamine levels (21). The performance in perceptual cognitive sensitivity judgments of healthy persons scoring high in MI was unaffected by an experimentally induced dopamine challenge. By contrast, low MI persons’ sensitivity decreased under the influence of a dopamine agonist. Moreover, with respect to their response behavior, low-scoring MI persons became less and high MI scorer more conservative under increased dopamine levels (21).

Up to our knowledge, there is no study examining whether and how often persons suffering from EE seek help, and how many of them approach the mental health system. A report by Hellmeister and Fach (22) for a committee of inquiry of the German government has described the complicated structures of motivations that compel help-seekers to approach unprofessional healers. It is important that the help given to people with EE is adequate, because the valence and interpretation of the EE could determine – among other things – whether EE can add to psychological health (3).

The way in which persons reporting EE seek help has up to now mostly been considered from the perspective of mental disorders. However, to infer a close interrelation seems to be premature, as the only systematic study known to cover this issue so far (3) concluded that persons seeking assistance because of EE did not necessarily suffer from mental disorder. Help-seeking caused by mental disorders has been analyzed extensively in large studies (23–25) that have found that only about 25–33% of individuals diagnosed with a mental disorder receive professional treatment. The most consistent predictor for help-seeking across different syndromes has been “subjective suffering” due to the disorder (26).

The aims of this study were to explore the quality and quantity of EE in the general population, to identify predictors of help-seeking because of EE, and to determine when the mental health care system is involved.

## Materials and Methods

### Sample

The sample of persons who received the on-line questionnaires represented the Swiss population in terms of gender, age, and education. Subjects were part of a pool from two professional recruiters, panelbiz^1^ and respondi^2^. The first recruiter provided a group of about 45,000 persons, from which a stratified quota sample of about 5000 persons was selected and asked for participation. Because this quota was incomplete, more persons were added from the pool of the first recruiter as well as others from the second recruiter. The individuals were initially invited with a standard-text email message containing formalities and technical details. Afterward, the on-line questions required approximately 15 min to answer, for which the participants received 5 Swiss Franks (4.1 Euro) as small compensation. The study was approved by the local ethics committee^3^.

### Instruments

Inquiries about EE were made via the PAGE-R (27), a revised and condensed version of PAGE (Fragebogen zur Erfassung der Phänomenologie Aussergewöhnlicher Erfahrungen/Questionnaire for the Assessment of the Phenomenology of EE) (28, 29) [for more details see Ref. (29, 30)]. The PAGE, created by the Institute for Frontier Areas of Psychology and Mental Health (IGPP), characterizes the phenomenology of EE according to the phenomenological classification indicated above. The revised version applied in this study had been developed recently, and can be used with on-line studies. The four factors in PAGE-R included external phenomenon (eight items), internal phenomenon (eight items), phenomenon of coincidence (eight items), and psychophysical dissociation (eight items). Possible answers were 0 = “never,” 1 = “rarely,” 2 = “sometimes,” 3 = “often,” or 4 = “very often.” Mean scores for these were calculated (Cronbach’s alpha = 0.886, 0.881, 0.895, and 0.884, respectively). Each section was supplemented by questions specifying when the phenomenon had occurred (during the last 12 months, last 5 years, last 10 years, longer ago than 10 years, or before the age of 18), as well as one question that assessed whether the person had been concerned with such a phenomenon up until now (0 = “not at all,” 1 = “a little,” 2 = “partly,” 3 = “fairly,” or 4 = “very”). Questions addressing the impact of the phenomenon were combined on one scale representing overall mean impact, and the questions on the location of the experiences within the person’s lifespan were combined on one scale representing the “density” of EE. The PAGE-R concluded with 14 items (0 = “not at all,” 1 = “a little,” 2 = “partly,” 3 = “fairly,” or 4 = “very”) specifying the circumstances (e.g., spontaneous, against one’s will) and causes (e.g., following life events, following contact with healer) of those experiences. A factor analysis^4^ of the 14 items yielded three factors – “experiences caused by spiritual techniques, esoteric practices, or contact with healers” (7 items, Cronbach’s alpha = 0.771); “experiences with negative valence” (7 items, Cronbach’s alpha = 0.759); and “spontaneous experiences with rather positive valence” (4 items, Cronbach’s alpha = 0.746), all of which were used in the analyses. At the end of the PAGE-R, two questions^5^ on help-seeking were added that differentiated among seeing either a healer, shaman, or psychic (further referred to as healers), or else a psychiatrist, psychotherapist, or psychologist. In addition, questions were asked about the socio-demographical attributes and diagnoses of mental disorders for the subject himself/herself and persons in his/her family. This included a preliminary question (yes/no/no answer) followed by an open question about the exact name of the disorder. The participants’ answers were classified as exactly as possible into broad categories.

Magical ideation was assessed via the MI (9) questionnaire. The individual degree of “vulnerability” for believing in MI can lead to a distinction between “sheep” (believers) and “goats” (skeptics) (31). Moreover, the two classes differ with regard to their success in experimental studies that have focused on extrasensory perception (31–33), associative tasks, semantic priming, and other cognitive processes (21, 34–37). The broadly used MI encompasses 30 items that can be answered with “yes” or “no.” After the MI scores were summed here (Cronbach’s alpha = 0.808), the median value was used to differentiate between “skeptics” and “believers.”

### Statistical analyses

Statistical analyses comprised two steps. First, any possible predictors of help-seeking were examined, using bivariate significance testing with a dependent variable that differentiated among seeing a healer, mental health professional, or both. Second, the bivariate significant variables were combined into one model that used multinomial logistic regression analysis to identify the strongest predictors of help-seeking. Analyses were performed with SPSS/PASW Statistics 18.

## Results

The final sample consisted of 1580 persons. Of the 32 cells in the quota table, 18 were entirely filled; the mean deviance from the desired number was 7.0%. The mean age was 39.1 years and 51.6% of the participants were women. In the education category, 11.5% (181) had completed compulsory school, 43.4% (686) received vocational training, 11.2% (177) had a high school diploma, and 33.9% (536) had finished university or another type of higher education (Table 2).

In total, 100 participants (7.4% of the 1353 participants who answered the questions on help-seeking) had asked for assistance from a healer, shaman, or psychic (“healer”), and 109 participants (8.1%) had sought help from a psychiatrist, psychotherapist, or psychologist (“mental health professional”). Of the participants answering the questions on help-seeking, 5.5% had seen only a healer, 1.8% saw both healer and mental health professional, 6.2% preferred a mental health professional, and 86.4% did not seek help because of their experiences. The rate of self-indicated actual and/or past diagnosis of mental disorder was 16.1% (3.2% no answer), while 22.2% (3.9% no answer) had indicated a history of psychiatric disorder(s) in the family. Table 1 shows the categories of diagnoses specified by the participants.

**Table 1:** **Self-reported diagnoses of participants**.

	Self		Family	
	*N*	%	*N*	%
No	1257	80.7	1169	74.0
No answer	50	3.2	61	3.9
None indicated	50	3.2	13	0.8
Depression	121	7.7	212	13.4
Anxiety disorders	29	1.8	11	0.7
Burnout	23	1.5	18	1.1
Bipolar disorder	8	0.5	12	0.8
Borderline personality disorder	8	0.5	6	0.4
ADHS	7	0.4	7	0.4
Substance misuse/dependence	7	0.4	6	0.4
Trauma/PTSD	4	0.3	0	0.0
Obsessive compulsive disorder	3	0.2	3	0.2
Somatoform/developmental disorder	3	0.2	5	0.3
Schizophrenia	2	0.1	45	2.8
Various, does not know name	2	0.1	9	0.6
Dementia	0	0.0	7	0.4
Others	16	1.0	26	1.6

*Psychiatric diagnoses indicated by the participants themselves, categorized along the main disorders. Multiple responses were possible*.

Depression and anxiety disorders were the most frequently mentioned by the participants themselves, followed by burnout, bipolar disorder, borderline personality, ADHD, and substance use (Table 1). Within the family, the most frequent disorder was depression followed by schizophrenia. The latter was reported at a quite frequent rate of 2.8%.

**Figure 1:**
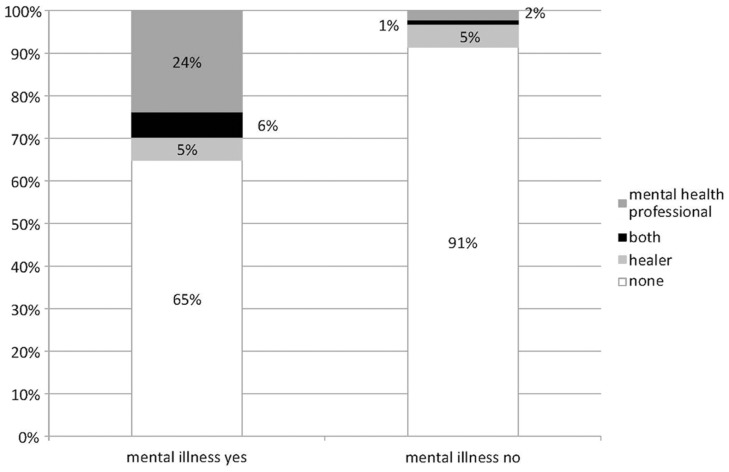
**Help-seeking because of exceptional experiences, differentiated by self-reported mental illness**.

Indicating a mental disorder (current or past) coincided with a tendency to visit both a healer and a mental health professional, or else a mental health professional alone, but not a healer alone (Figure 1). Persons with diagnosis had more often sought help (35.1%) than persons without (8.6%).

Of all the participants, the majority reported having at least one EE during their lifetime. A “no” answer was given by 141 persons (8.9%) to all PAGE-R items about EE. Approximately 25% denied all items from Class 1 “external phenomenon” and Class 2 “internal phenomenon,” whereas about 20% acknowledged experiencing Class 3 “phenomenon of coincidence” and 50% were in Class 4 “psychophysical dissociation.” Experiences within Classes 1 and 2 (mean 0.76) were rated as less frequent than within Class 3 (0.96) but more frequent than Class 4 (0.55). The mean impact was quite low (between “not at all” and “a little”), and most phenomena occurred during only one phase of life (mean = 1.14) (Table 3). MI was also quite frequent. According to the MI, only 11 participants answered all questions with “no.” The median was 9 positive answers (maximum 25) – individuals above that were classified as believers, those below as skeptics (Table 2).

**Table 2:** **Categorical predictors of lifetime help-seeking because of EE (*N* depending on variable)**.

	Total		Healer		Psychiatrist	
	*N*	%	*N*	%	*N*	%
Total	1580	100	100	7.4	109	8.1
Gender						
Woman	816	51.6	59	8.2	52	7.3
Man	764	48.4	41	6.4	57	8.9
Education						
Compulsory school	181	11.5	**24**	**15.5**	**22**	**14.2**
Vocational training/school	686	43.4	**38**	**6.6**	**48**	**8.4**
Secondary school	177	11.2	**14**	**8.8**	**6**	**3.8**
University/higher education	536	33.9	**24**	**5.2**	**33**	**7.1**
Marital status						
Unmarried	644	40.8	**37**	**6.6**	**36**	**8.2**
Married	694	43.9	**36**	**6.1**	**48**	**6.5**
Divorced	225	14.2	**23**	**12.2**	**22**	**11.7**
Widowed	17	1.1	**4**	**28.6**	**3**	**21.4**
Partner
No	436	27.6	32	8.6	35	9.4
Yes	1144	72.4	68	6.9	74	7.5
Children
No	745	47.2	46	7.2	50	7.8
Yes	835	52.8	54	7.6	59	8.3
Living alone
No	1087	68.8	**59**	**6.3**	**66**	**7.0**
Yes	493	31.2	**41**	**9.9**	**43**	**10.4**
Occupation
School	123	7.8	**4**	**3.8**	**6**	**5.7**
Employed	1173	74.2	**74**	**7.5**	**67**	**6.8**
Domestic work	224	14.2	**12**	**6.0**	**18**	**9.0**
Pensioner	60	3.8	**10**	**12.7**	**18**	**31.0**
Cultural background
Middle Europe	1469	93.0	90	7.1	100	7.9
Other	111	7.0	10	10.0	9	10.2
Denomination
Protestant	504	31.9	23	5.3	32	7.4
Catholic	469	29.7	36	8.7	34	8.3
Other	114	7.2	9	9.5	7	7.4
None	493	31.2	32	7.7	36	8.7
Belong to a community of faith
Yes	471	29.8	28	6.9	31	7.6
No	1109	70.2	72	7.6	78	8.3
Believing
Yes	882	55.8	**83**	**10.6**	69	8.8
No	698	44.2	**17**	**3.0**	40	7.0
Self-reported diagnosis – self
Yes	225	16.1	**27**	**11.3**	**71**	**29.7**
No	1275	80.7	**69**	**6.5**	**34**	**3.2**
No answer	50	3.2	**4**	**8.9**	**4**	**8.9**
Self-reported diagnosis – family
Yes	350	22.2	24	7.5	**37**	**11.5**
No	1169	74.0	75	7.7	**64**	**6.5**
No answer	61	3.9	1	1.9	**8**	**15.4**
MI
Skeptics	827	52.3	**11**	**1.7**	**34**	**5.3**
Believers	753	47.7	**89**	**12.5**	**75**	**10.5**

*Within a category, bolded figures are significantly different at *p* < 0.05, according to Chi-square tests*.

Tables 2 and 3 indicate the bivariate associations between predictors and help-seeking. Significant interrelations were found for education, marital status, living alone, occupation, believing, (history of) psychiatric diagnosis for self or family, MI skeptics and believers, and all PAGE-R indicators.

**Table 3:** **Continuous predictors of lifetime help-seeking because of EE (*N* depending on predictors and help-seeking variables)**.

	Total		Healer				Psychiatrist			
			Yes		No		Yes		No	
	*N*	Mean	*N*	Mean	*N*	Mean	*N*	Mean	*N*	Mean
Age	1580	39.09	100	39.80	1253	38.81	109	40.81	1244	38.71
PAGE-R external phenomenon (mean)	1173	0.76	**100**	**1.37**	**1041**	**0.71**	**100**	**0.82**	**1041**	**0.62**
PAGE-R internal phenomenon (mean)	1165	0.76	**98**	**1.38**	**1021**	**0.73**	**101**	**0.81**	**1018**	**0.61**
PAGE-R phenomenon of coincidence (mean)	1291	0.96	**99**	**1.73**	**1141**	**0.92**	**103**	**0.83**	**1137**	**0.72**
PAGE-R psychophysical dissociation (mean)	776	0.55	**88**	**0.96**	**663**	**0.51**	**87**	**0.71**	**664**	**0.55**
PAGE-R induced by spiritual techniques	1353	0.58	**100**	**1.40**	**1253**	**0.51**	**109**	**0.95**	**1244**	**0.55**
PAGE-R negative valence	1353	0.81	**100**	**1.25**	**1253**	**0.77**	**109**	**1.55**	**1244**	**0.74**
PAGE-R spontaneous, positive valence	1353	1.53	**100**	**1.94**	**1253**	**1.50**	109	1.67	1244	1.52
PAGE-R density (mean)	1439	1.14	**100**	**1.74**	**1253**	**1.17**	**109**	**1.44**	**1244**	**1.20**
PAGE-R impact (mean)	1439	0.80	**100**	**1.92**	**1253**	**0.76**	**109**	**1.31**	**1244**	**0.81**
MI (mean)	1580	0.32	**100**	**0.51**	**1253**	**0.33**	**109**	**0.42**	**1244**	**0.33**

*Within a category, bolded figures are significantly different at *p* < 0.05, according to *t*-tests*.

Table 4 presents the bivariately significant predictors in a multinomial regression with the dependent variable, combining both healer and mental health professional.

**Table 4:** **Multinomial regression with predictors of help-seeking because of EE**.

		Healer		Both		Mental health professional	
		*p*	Exp (*B*)	*p*	Exp (B)	*p*	Exp (*B*)
Age		0.304	0.986	0.966	0.999	0.115	1.023
PAGE-R external phenomenon		0.544	1.189	0.976	1.015	0.202	0.605
PAGE-R internal phenomenon		0.849	1.069	0.666	0.772	0.156	1.722
PAGE-R phenomenon of coincidence		0.340	0.753	0.893	0.927	0.359	0.732
PAGE-R psychophysical dissociation		0.660	0.862	0.914	0.943	0.508	0.763
PAGE-R induced by spiritual techniques		**0.000**	**3.249**	**0.000**	**6.651**	0.720	0.888
PAGE-R negative valence		**0.007**	**1.965**	**0.006**	**3.261**	**0.000**	**8.043**
PAGE-R spontaneous, positive valence		**0.011**	**0.553**	**0.005**	**0.267**	**0.000**	**0.297**
PAGE-R impact		**0.000**	**2.046**	**0.015**	**2.462**	0.057	1.636
PAGE-R density		0.236	1.178	0.555	1.181	0.966	0.992
Gender	Man	**0.011**	**0.443**	**0.038**	**4.064**	0.884	1.047
	Woman						
Education	Compulsory	**0.028**	**2.196**	0.544	1.487	0.991	1.005
	Other						
Believing	Yes	**0.003**	**2.898**	0.198	2.318	0.292	0.721
	No						
Marital status	(Un)married	0.592	0.810	0.202	0.428	0.241	1.678
	Divorced/widowed						
Living situation	No	0.292	0.719	0.142	0.468	0.473	0.793
	Yes						
Occupation	Other	0.956	0.959	**0.044**	**0.243**	0.975	0.983
	Pensioner						
Self-reported	Yes	0.367	0.702	**0.021**	**3.697**	**0.000**	**13.141**
Diagnosis self	No						
Self-reported	Yes	0.835	0.934	0.243	0.439	0.100	1.666
Diagnosis family	No						
MI	Skeptic	**0.028**	**0.370**	0.809	0.838	0.329	0.712
	Believer						

*Dependent variable = healer/mental health professional/both/none (reference). *N* = 1270 for sample of participants who answered the questions concerning help-seeking and psychiatric diagnoses. Within a category, bolded figures are significant at *p* < 0.05. Pseudo *R*-square: Cox and Snell = 0.312, Nagelkerke = 0.480, McFadden = 0.316*.

Help-seeking was predicted by the additional attributes of EE rather than by the classes of experiences measured by the PAGE-R questionnaire. When experiences were induced by (spiritual) techniques, and when they had more impact, individuals were more likely to visit a healer or both a healer and a mental health professional. Seeing the latter or both was predicted only by diagnosis. Negative valence triggered help-seeking in general. Skeptics, as determined via the MI, sought help less often from a healer alone.

Some socio-demographical variables clearly predicted help-seeking behavior in the combined model. For example, compared with men, women visited more often a healer alone but less often both a healer and mental health professional. Believing in a deity/less education also were associated with help-seeking from a healer alone. Persons who were not employed visited both more often.

Table 5 illustrates that persons with a self-reported diagnosis of mental disorder four times more often sought help, even when all characteristics of EE were controlled for. The relation between mental disorder and help-seeking remained highly significant in the model with number of different EE, mean frequency of EE, frequency over the life course (density), additional characteristics of EE, and mean burden caused by the EE.

**Table 5:** **Logistic regression including characteristics of. EE**.

	*B*	SD	Wald	df	*p*	Exp (*B*)
Diagnosis of mental disorder (yes)	1.493	0.207	52.136	1	0.000	4.452
PAGE-R number of EE	0.066	0.027	6.152	1	0.013	1.068
PAGE-R mean frequency of EE	−1.134	0.411	7.636	1	0.006	0.322
PAGE-R induced by spiritual techniques	0.110	0.027	16.131	1	0.000	1.117
PAGE-R negative valence	0.166	0.024	47.482	1	0.000	1.180
PAGE-R spontaneous, positive valence	−0.222	0.038	34.098	1	0.000	0.801
PAGE-R impact	0.921	0.144	40.928	1	0.000	2.513
PAGE-R density over life course	0.091	0.103	0.785	1	0.376	1.096

*This regression illustrates that persons with a self-reported diagnosis of a mental disorder seek more often help than those without, controlling for all possible characteristics of EE. *N* = 1308 for sample without missing values. Pseudo *R*-square: Cox and Snell = 0.203, Nagelkerke = 0.371*.

## Discussion

In this sample from the Swiss general population, almost all participants (91%) indicated having had at least one exceptional experience. The typology of EE did not correlate with self-reported mental disorder. This was rather unexpected, because an earlier study had shown that persons with an internal phenomenon have contact with the psychiatric field more often (3). The rate of EE-induced help-seeking was 13.6% among the persons with at least one EE. About 8.1% thereof visited a mental health professional, 7.4% approached a healer. Help-seeking because of EE was globally associated with negative valence of EE. Help-seeking was less frequent in persons without a self-reported mental disorder (8.6%) than in persons with such a disorder (35.1%) (OR = 5.7). But interestingly, even when the frequency, the valence, and other attributes of EE were controlled for, people without a disorder sought four times less often help because of EE than persons with a disorder (OR = 4.5). Self-reported mental disorder was highly correlated with the rate of EE-induced help-seeking in the mental health care system, but not with alternative sources of help: independent of mental disorder, about 5% of the participants visited healers, shamans, or psychics because of EE. Multinomial regression revealed a preference for healers in women with less education, who described themselves as believing (according to a global question and according to the MI) and also having had more impressive EE. Other predictors of help-seeking besides gender and education were occupation and the trigger of the experienced EE.

### Connection mental disorder – EE – help-seeking

The frequency of EE (91%) and self-reported mental disorder (16%) in the examined population is comparable with other studies (1, 2, 38). Therefore, EE are too frequent to be an unequivocal indicator of a mental disorder. The subjective burden that might lead one to seek help is according to psychopathology not indicative of the differentiation between illness and health. Whether this is the case in EE remains to be determined. However, it has been shown that if EE are successfully coped with, they can add to psychological health (3). Help-seeking in general is more frequent when experiences are of rather negative valence. It has been shown that the valence of EE also determines whether experiences could contribute to mental health (3). Therefore, helping persons to integrate their experiences and assign meaning to them, which should lead to a more positive valence (37, 39), might not only be beneficial in the case of (comorbid) mental disorder but also for healthy individuals. When persons with such a diagnosis – and also their assistants – automatically subsume EE under the symptoms of that disorder, they might miss these positive coping strategies. As Belz-Merk and Fach (3) have stated, the mental health care system might not be entirely adequate for persons with EE. If this is the case, an adaptation might be necessary to optimally help people with EE, especially those without concomitant mental disorder. It is important that the mental health system meets needs of people with EE because firstly, many of them seek help from the mental health care system, and secondly, because there is some evidence that a therapy tailored to enhance coping with EE could be beneficial for people with and without mental disorder.

### Help-seeking and self-reported mental disorder

Our data indicate that people suffering from EE but without self-reported mental disorder less often sought help even when attributes of EE, including the frequency and valence, were controlled for. We do not know whether persons *without* self-reported mental disorder do require less help to cope with their EE than persons with a disorder. It cannot be determined from our data whether persons who seek help suffer from prodromal or initial psychiatric disorders. We explain the lower rates of help-seeking by a higher threshold to seek help from the mental health care system in persons without mental disorder. Possibly one aspect of the high threshold is that it is difficult to approach that system without declaring oneself as mentally ill. Whether there is an unmet medical need in mentally healthy persons with EE or whether there is an alternative explanation for the lower rates of help-seeking should be confirmed by further research, especially longitudinal studies assessing EE, mental disorder, the degree of suffering, and concomitant factors as resilience, coping, and help-seeking are needed.

The higher rate of help-seeking in persons with (a history of) self-reported mental disorder is possibly explained by the *a priori* involvement that persons suffering from mental disorder had with the mental health treatment system. Because their confidants are already mental health professionals, those contacts are then re-activated because of EE. A second possibility is that those with a self-reported diagnosis of mental disorder have another system of beliefs to explain feelings and perceptions they do not understand at first sight drawing on a “disease” model. This hypothesis parallels the supposition that those without a self-reported mental disorder but seeking help from a healer have a spiritual view of EE. The third explanation for the higher rate of help-seeking is that persons who sought help had a higher level of subjective distress, which was not assessed by the questionnaires used in this study.

### Other predictors of help-seeking

In our combined model, socio-demographical predictors were gender, education, and occupation. Women with lower education, being a believer rather than a skeptic according to the MI, and describing themselves as believing in some deity, more often sought help from a healer alone because of EE. Gender has been associated quite consistently with help-seeking in persons with mental disorders, with women having higher treatment rates (24). In contrast, our data indicated that gender modulated the choice of assistant, with women visiting a psychiatrist less often than men. That women with less education visited more often healers than other sources of help indicates a higher acceptance of healers. Whether this implies any risk remains to be determined. Our results are in line with other fields of research that show a therapeutic undersupply of women (40). Interestingly, men who described themselves as pensioners or benefit recipients belonged to the group of people who visited both a healer and a mental health professional. However, this result can be misleading because the question about occupation did not assess unemployment. Therefore, we do not know whether unemployed persons selected “homework” or “pensioner, receiving benefit” as their occupational status. In the previous IGPP study on help-seeking, persons having contact with psychiatry were 48% male, and 63% were not working (3). Unfortunately, our cross-sectional data did not allow us to disentangle further the causal interrelations among mental health status, occupation, gender, and EE.

Another interesting predictor of help-seeking was MI. Importantly, EE and MI are not the same. Persons may highly believe in the paranormal without having the respective experiences, and highly skeptical persons may experience things they do not believe in. In this study, skeptics were less inclined to seek help from a healer alone, even if EE were controlled for. This finding supports the hypothesis that systems of belief guide the choice of the helping agent.

In general, negative valence of EE was associated with help-seeking. When EE had more impact (i.e., higher scores on the item “How much are you concerning yourself with your experiences up to now?”), persons preferred seeing a healer or both sources of help. The involvement of a healer could be explained that persons who were more focused on their EE sought for an explanation that a professional psychotherapy was not able to give. Another predictor for help-seeking from a healer or both sources was the trigger of EE, e.g., the experiences were induced by spiritual techniques. Therefore, persons already familiar with esoteric practices were more prepared to seek help within their corresponding system of belief. Exceptional experiences can be triggered in manifold ways: Often they follow a crisis situation or an event such as death within an individual’s environment. But they can also occur in association with altered states of consciousness either during the transition between wakefulness and sleep or when induced by psychotropic drugs. Other stimulating situations include involvement in meditation, spiritual, occult or magical practices; contact with healers, shamans, or mediums; or intense psychotherapy or psychoanalysis. Therefore, it remains unclear whether the contact with healers triggered the EE, or whether people activated previous contacts to healers to discuss their EE.

### Conclusion

Our results indicate that persons with EE who do not suffer from self-reported mental disorder less often sought help because of EE. We attributed this to a high inhibition threshold for people without a history of self-reported mental disorder to seek professional help. Especially, less educated women did not approach the mental health care system as often as other persons with EE, but preferred seeing a healer. There are some hints that an adequate therapy might be beneficial in the case of EE. Whether not seeking help or preferring a healer is of negative consequences remains to be determined. The higher rate of help-seeking from the mental health care system in persons with acute or previous mental disorder leads to the assumption that either pre-existing contact, or the dominant system of belief of the person, led help-seeking. A parallel preference for healers in persons with a spiritual worldview supports this interpretation. Whether subjective distress also led to higher rates of help-seeking remains to be determined.

### Limitations

It remains to be verified whether the mental health care system meets the needs of persons with EE or whether a special treatment system might be required, as Belz-Merk and Fach (3) suggested.

It is unclear how the readiness of individuals to reveal EE can be faithfully assessed. Although there may be considerable openness for experiences and the willingness to admit them by the general population, a tendency to rationalize those experiences and include narrative caveats in official reports can invite caution (1).

The sample we used represented the Swiss general population based only upon gender, age, and level of education. Even if the total sample size was considerable, cell sizes of statistical analyses sometimes were small, so the detailed results need confirmation by additional studies. Diagnoses of mental disorder were assessed by directly asking the respondents, but were not verified through a diagnostic interview. The questions did not determine whether the reported EE had occurred unequivocally as a symptom of a mental disorder. Diagnoses, EE, and help-seeking behavior were assessed according to lifetime prevalence. Therefore, association could not implicate contemporaneity. While the PAGE-R assessing EE was quite comprehensive, the MI covered only a small fraction of their overall spectrum.

## Author Contributions

Karin Landolt analyzed and interpreted the data and drafted the manuscript. Amrei Wittwer contributed to the study design, interpreted the data, and drafted the manuscript. Thomas Wyss and Lui Unterassner contributed to the study design and the acquisition of data and revised the manuscript critically. Wolfgang Fach contributed to the conception and design of the study and was involved in drafting the manuscript. Peter Krummenacher, Peter Brugger, Helene Haker, Wolfram Kawohl, Pius August Schubiger, Gerd Folkers, and Wulf Rössler contributed to the conception and design of the study and revised the manuscript critically. All authors have given final approval for the version to be published and agreed to be accountable for all aspects of the work.

## Conflict of Interest Statement

The Associate Editor Alexandre Andrade Loch declares that, despite being affiliated to the same institution as author Wulf Rössler, the review process was handled objectively and no conflict of interest exists. The authors declare that the research was conducted in the absence of any commercial or financial relationships that could be construed as a potential conflict of interest.

## Footnotes

^1^www.panelbiz.ch

^2^www.respondi.com

^3^Kantonale Ethikkommission Zürich, www.kek.zh.ch

^4^Principal Component Analysis, Varimax-Rotation with Kaiser-Normalisation, converged in six iterations; factor-loadings of variables were interpreted when higher than 0.3.

^5^(a) “(those experiences) led me to seek help from a psychiatrist, psychotherapist, or psychologist” (yes/no). (b) “(those experiences) led me to seek help from a shaman, healer, or psychic” (yes/no).
